# Post-traumatic headache after mild traumatic brain injury in a one-year follow up study – risk factors and return to work

**DOI:** 10.1186/s10194-022-01398-9

**Published:** 2022-02-19

**Authors:** Yvonn Kraemer, Kaisa Mäki, Ivan Marinkovic, Taina Nybo, Harri Isokuortti, Antti Huovinen, Antti Korvenoja, Susanna Melkas, Hanna Harno

**Affiliations:** 1 Department of Neurology, Clinical Neurosciences, Helsinki University Hospital and University of Helsinki, Helsinki, Finland; 2 Department of Neurology, Helsinki University Hospital, University of Helsinki, Haartmaninkatu 4, P.O.Box 340, 00029 Helsinki, Finland; 3 Division of Neuropsychology, HUS Neurocenter, Helsinki University Hospital and University of Helsinki, Helsinki, Finland; 4 Department of Neurosurgery, Clinical Neurosciences, Helsinki University Hospital and University of Helsinki, Helsinki, Finland; 5 HUS Diagnostic Centre, Radiology, University of Helsinki and Helsinki University Hospital, Helsinki, Finland

**Keywords:** Risk factor, Brain injury, mTBI, Post-traumatic, Headache, PTH

## Abstract

**Background:**

Post-traumatic headache (PTH) is a common symptom following mild traumatic brain injury (mTBI). Patients at risk to develop acute PTH (aPTH) and further persistent PTH (pPTH) need to be recognized.

**Methods:**

This is a one-year follow-up of 127 patients with mTBI, aged 18 to 68, referred to outpatient clinic in the Helsinki University Hospital. Symptoms were assessed at the emergency department (ED), with structured interview at outpatient clinic visit and with Rivermead post-concussion symptom questionnaire at one, three, and 12 months after injury. Psychiatric disorders were assessed with Structured Clinical Interview for DSM-IV Axis I disorders at 3-4 months and return to work (RTW) from patient records.

**Results:**

At one month, 77/127 patients (61%) had aPTH. According to multiple logistic regression analysis, risk factors for aPTH were headache at the emergency department (ED) (OR 5.43), other pain (OR 3.19), insomnia (OR 3.23), and vertigo (OR 5.98). At three months, 17 patients (22% of aPTH patients) had developed pPTH, and at one year, 4 patients (24% of pPTH patients) still presented with pPTH. Risk factors for pPTH at three months were older age (OR 1.06) and current insomnia (OR 12.3). The frequency of psychiatric disorders did not differ between the groups. pPTH patients performed worse on their RTW.

**Conclusions:**

Risk factors for aPTH were insomnia, headache at ED, other pain, and vertigo and for pPTH, insomnia and older age. RTW rate was lower among pPTH patients.

## Introduction

Traumatic brain injury (TBI) is estimated to affect 69 million individuals per year [1]. Mild TBI (mTBI) represents 70-90% of all TBIs [2–4]. Post-traumatic headache (PTH) is one of the most common symptoms following TBI [5–7] and is more common after mTBI than moderate or severe TBI [8].

Typically patients with mTBI recover within days or weeks. However, a subgroup of patients experience prolonged post-traumatic symptoms including headache, which can cause long-term disability and delayed return to work [9–11].

By definition, the onset of PTH occurs within seven days following the head trauma, within seven days after recovering from unconsciousness, or within seven days after recovering the ability to report symptoms [12]. During the first three months from onset, PTH is defined as acute, and beyond that, persistent [12].

The nature of PTH varies. Some studies have discovered that most people with PTH have a migraine-like headache [5], whereas others have found a higher prevalence of tension-type headaches [13]; still others experience features of chronic migraine- or cluster-like phenotypes [14, 15].

The difference of migraine and pPTH has previously been studied. E.g. brain functional neuroimaging findings and calcitonin gene-related peptide (CGRP) levels differ in migraine compared with pPTH suggesting distinct pathophysiologies. On the other hand, there might also be shared pathophysiology, of which initial open label studies on CGRP monoclonal antibodies in the treatment of pPTH have shown preliminary evidence [16].

According to previous studies, female gender may be a risk factor for PTH [10, 17], although there is also conflicting evidence [5].  Additionally, younger age, previous headache, and headache immediately after the injury are risk factors for PTH [10, 17]. Individuals with PTH often report concomitant symptoms such as anxiety, depression, and sleep disturbance [10, 18]. Psychiatric comorbidities are complex and prevalent also in migraine and shared pathophysiology mechanisms may play a key role in headache chronification. These are important to consider in the diagnostic work up and treatment [19].

According to a prognostic model developed for clinicians, lower education, injury severity, and psychological factors such as maladaptive coping style and pre-injury mental health problems play a role for prolonged symptoms in mTBI [20]. However, specific risk factors for development of persistent PTH (pPTH) remain understudied.

We aimed to thoroughly identify potential risk factors for acute PTH (aPTH) and further pPTH in mTBI patients by utilizing the Rivermead Post Concussion Symptom Questionnaire (RPQ) [21], Structured Clinical Interview for DSM-IV Axis I disorders (SCID-I) [22], and patient records from the emergency department (ED) and clinical neurology outpatient visits within one year’s follow-up.

We hypothesized that previous primary headaches, other pain condition, sleep disorders, and psychiatric burden would act as a risk factors for acute- and persistent PTH. Further, we presumed that pPTH would have negative impact on RTW.

## Methods

### Study design and patients

This is a substudy of a prospective cohort study conducted at the Traumatic Brain Injury Outpatient Clinic in the Helsinki University Hospital from March 2015 until September 2018 [23] Inclusion criteria were consecutive patients with mild brain injury and post-traumatic headache according to International Classification of Headache Disorders (ICHD-3) criteria [12], Finnish as their native language, aged 18 to 68 years. Exclusion criteria were alcohol or drug dependence, previously diagnosed schizophrenia or schizoaffective disease, mental retardation, and vision or hearing disability. The study was approved by the local ethical board of the Helsinki University Hospital. All patients gave their written informed consent.

Patients underwent 3T magnetic resonance imaging (MRI) within approximately two weeks after incident TBI. A senior neuroradiologist evaluated all MRI scans.

World Health Organization (WHO) criteria of mild traumatic brain injury were used [24]. WHO criteria include (1) one or more of the following: confusion or disorientation, loss of consciousness for 30 min or less, post-traumatic amnesia for less than 24 h, or other transient neurological abnormalities, such as focal signs, seizure, and intracranial lesion not requiring surgery; and (2) Glasgow Coma Scale (GCS) score of 13-15 after 30 min post-injury or later upon presentation for health care. WHO criteria approve minor trauma findings in CT or MR imaging.

In the study aPTH and pPTH were assigned according to ICHD-3 criteria [12]. Presence of headache was documented first at the emergency department (ED); aPTH was defined as headache occurring within seven days from the TBI (based on documents from the ED and structured interview together with RPQ at one month). At three months (pPTH) and 12 months after the injury, headache and other symptoms were assessed with Rivermead Post-Concussion Symptoms Questionnaire (RPQ), a 16-item self-report questionnaire measuring the presence and severity of common post-concussion symptoms on a 5-point Likert Scale (0-4) [21]. In our analysis the total RPQ score also included scale 1 (no longer a problem). If patients had not reported headache at one month, but reported it at three months, it was not classified as pPTH. Characteristics of pPTH were collected from patient records from the clinical neurologist appointment.

Questions regarding insomnia (yes/no) and other pain (yes/no) were part of the structured interview at one month.

At three to four months after injury, psychiatric disorders were assessed with the Structured Clinical Interview for DSM-IV Axis I disorders (SCID-I) [22].

Return to work (RTW) data was collected retrospectively from the patient records and evaluated with one-day accuracy. Individuals who returned to part-time work were also taken into account. We regarded a return to studies for full-time students as RTW.

**Fig. 1:**
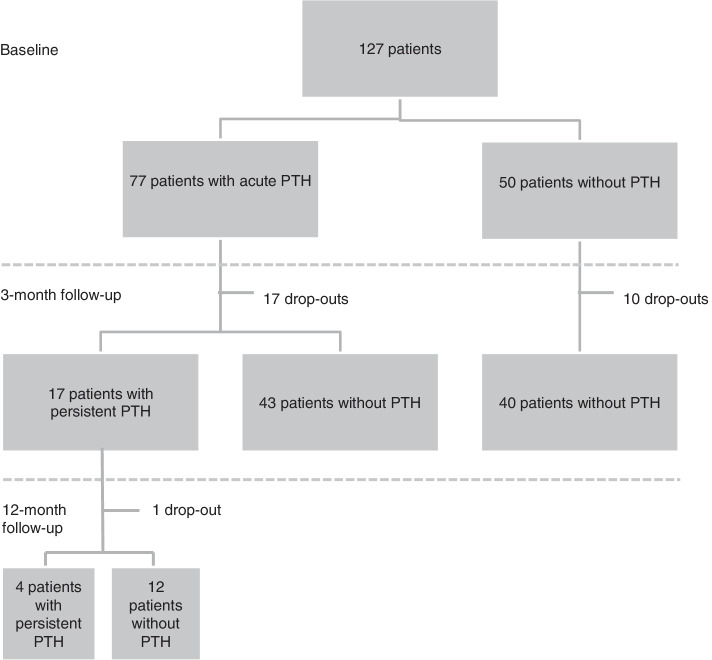
Distribution of patients according to presence of post-traumatic head-ache (PTH)

We recruited 131 patients with mTBI and 127 of them had filled in information about headache at one month. The study cohort consisted of 127 patients. At three months, information about headache was available for 100 patients and at 12 months, for 72 patients. The patient flow chart is illustrated in Fig. 1.

We named those patients who had acute PTH at one month aPTH and those patients who had acute PTH but not persistent PTH recovered-aPTH (r-aPTH).

### Statistical analyses

Continuous variables are presented as mean (standard deviation, SD) if normally distributed and as median (interquartile range, IQR) if not normally distributed. Categorical variables are given as frequencies and percentages. For group comparisons, Pearson’s chi-square was used for binary categorical variables, one-way ANOVA for normally distributed continuous variables, and nonparametric Mann-Whitney U-tests for non-normally distributed continuous variables. Univariate and multiple logistic regression analysis were used to assess the possible risk factors for PTH. We considered p-values <0.05 as statistically significant. IBM SPSS Statistics 24 was used to perform the analysis.

## Results

### Demographics

The mean age of the 127 patients was 40 years (SD 12.9) and 46.5% were female. One hundred fifteen patients (90.6%) were employed or full-time students before trauma. Ground-level falls were the most frequent cause for TBI (33%); 24 patients (18.9%) were under influence of alcohol at the time of the trauma, according to patient records.

The following minor traumatic brain lesions were found in MRI: 48 patients (37.0%): any traumatic intracranial lesion, acute subdural hemorrhage in 20 patients (15.7%), small epidural hematoma in 1 patient, traumatic subarachnoidal hemorrhage in 19 patients (12.6%), traumatic intracerebral hemorrhage in 5 patients (3.1%), and microhemorrhages indicating diffuse axonal injury in 26 patients (20.5%), and minor cerebral contusion in 14 patients (11.0%). None of the patients with trauma findings needed surgery.

### Patients with aPTH

At one month, 77/127 (61%) patients reported aPTH. Patient demographics for patients with aPTH and non-PTH are shown in Table 1.

**Table 1:** Patient demographics in aPTH and non-PTH patients one month after injury

Variable	aPTH		Non-PTH		aPTH vs. non-PTH
	Valid n		Valid n		p value
Female sex, n (%)	77	36 (46.8)	50	23 (46.0)	0.934
Age in years, mean (SD)	77	41.2 (13.9)	50	37.5 (11.3)	0.110
Education in years, mean (SD)	77	15.6 (4.4)	50	16.1 (3.3)	0.516
Previous migraine, n (%)	77	18 (23.4)	49	3 (6.1)	**0.011**
Previous other headache, n /(%)	77	35 ( 45.5)	49	17 (34.7)	0.232
Previous depression, n (%)	55	6 (10.9)	36	5 (13.9)	0.747
Previous insomnia, n (%)	77	31 (40.3)	50	10 (20.0)	**0.017**
Previous use of analgesic drug, n (%)	77	15 (19.5)	50	6 (12.0)	0.268
Traumatic lesion in MRI, n (%)	77	27 (35.1)	50	20 (40.0)	0.574
Wounding skull/face, n (%)	76	65 (85.5)	50	45 (90.0)	0.461
PTA at ED, n (%)	77	66 (85.7)	50	44 (88.0)	0.712
TLOC, n (%)	77	53 (68.8)	50	33 (66.0)	0.739
Headache at ED, n (%)	77	74 (96.1)	49	39 (78.0)	**0.003**
RPQ median (IQR)	66	13 (6.75-19.00)	43	4 (1.00-9.00)	**<0.001**
Other pain after injury, n (%)	76	47 (61.0)	49	16 (32.0)	**0.001**
Insomnia after injury, n (%)	77	47 (61.0)	50	13 (26.0)	**<0.001**
Vertigo after injury, n (%)	77	45 (58.4)	50	9 (18.0)	**<0.001**

Continuous variables: one-way ANOVA; dichotomous variables: Pearson Chi–square test

*aPTH* acute post-traumatic headache; *non-PTH* no post-traumatic headache; *PTA* post-traumatic amnesia (retro- and/or anterograde); *ED* emergency department; *LOC* loss of consciousness; *RPQ* The Rivermead Post Concussion Symptoms Questionnaire

Patients with aPTH had previous migraine (*p* = 0.033) and insomnia (*p* = 0.017) significantly more often than non-PTH patients. There was no difference in frequency of trauma findings between aPTH and non-PTH patients (Table 1).

Patients with aPTH reported headache significantly more often at the emergency department (ED) than non-PTH patients (*p* = 0.003). The median RPQ score at one month postinjury was significantly higher in aPTH than non-PTH patients (*p* < 0.001) (Table 1).

In multiple logistic regression analysis, headache at the ED (*p* = 0.029), other pain (*p* = 0.010), insomnia (*p* = 0.010), and vertigo (*p* < 0.001) were independent risk factors for aPTH (Table 2).

**Table 2:** Logistic regression analysis of the risk factors of acute post-traumatic headache

Factor	Odds ratio	95% Cl	P value
n = 127			
Headache at the ED	5.42	1.19-24.73	0.029
Other pain after injury	3.20	1.31-7.78	0.010
Insomnia after injury	3.23	1.33-7.89	0.010
Vertigo after injury	5.99	2.33-15.40	<0.001

*ED* emergency department; *Cl* Confidence Intervals

### Patients with pPTH

17/77 of aPTH patients (22%) developed pPTH. The mean age of pPTH patients was 44.3 years (SD 12.8) with 47% women. Previous self-reported migraine was in 3/17 of pPHT patients (18%), previous other headache in 8/17 (47%), and previous insomnia in 7/17 (41%). Trauma findings in MRI were detected in 10/17 (59%) of pPTH patients.

In comparison between pPTH (n = 17) and r-aPTH (n = 43) patients, no statistically significant differences were found in demographic or clinical characteristics (Table 3) including insomnia after injury (77% vs. 51%, p = 0.073), trauma findings in MRI (59% vs. 33%, p = 0.061), and previous migraine (18% vs. 26%, p = 0.737).

**Table 3:** Patient demographics and characteristics in patients with persistent and recovered-acute post-traumatic headache three months after injury

Variable	pPTH		r-aPTH		pPTH vs. r-aPTH
	Valid n		Valid n		P value
Female sex, n (%)	17	8 (47.1)	43	21 (48.8)	0.901
Age in years, mean (SD)	17	44.3 (12.8)	43	42.2 (14.6)	0.604
Education in years, mean (SD)	17	16.4 (3.9)	43	15.9 (4.9)	0.722
Previous migraine, n (%)	17	3 (17.6)	43	11 (25.6)	0.737
Previous other headache, n (%)	17	8 (47.1)	43	17 (39.5)	0.594
Previous depression, n (%)	17	2 (11.8)	37	4 (10.8)	1.000
Previous insomnia, n (%)	17	7 (41.2)	43	15 (34.9)	0.649
Previous use of analgesic drug, n (%)	17	3 (17.6)	43	8 (18.6)	1.000
Traumatic lesion in MRI, n (%)	17	10 (58.8)	43	14 (32.6)	0.061
Wounding skull/face, n (%)	17	15 (88.2)	42	36 (85.7)	1.000
PTA at ED, n (%)	17	13 (76.5)	43	38 (88.4)	0.256
TLOC, n(%)	17	10 (58.8)	43	31 (72.1)	0.319
Headache at ED, n (%)	17	17 (100)	43	41 (95.3)	1.000
RPQ median at three months (IQR)	17	21 (12.00-26.50)	43	2 (0.00-7.00)	<0.001
Other pain after injury, n(%)	17	9 (56.3)	43	29 (67.4)	0.425
Insomnia after injury, n (%)	17	13 (76.5)	43	22 (51.2)	0.073
Vertigo after injury, n (%)	17	8 (47.1)	43	25 (58.1)	0.437

Continuous variables: one-way ANOVA; dichotomous variables: Pearson Chi–square test

*pPTH* persistent post-traumatic headache; *aPTH* acute post-traumatic headache; *PTA* post-traumatic amnesia (retro- and/or anterograde); *ED* emergency department; *TLOC* transient loss of consciousness; *RPQ* The Rivermead Post Concussion Symptoms Questionnaire score; *IQR* Interquartile range

When comparing pPTH with non-PTH (n = 40) patients, differences were more pronounced. Non-PTH patients were younger (mean age 36.5 years, p = 0.026), and pPTH patients reported insomnia after injury significantly more often than non-PTH patients (76.5% vs. 22.5%, p < 0.001).

In three-month follow-up RPQ scores, even when headache was excluded from the total score, pPTH patients had significantly more concomitant symptoms compared with r-aPTH and non-PTH patients. The median RPQ score of pPTH patients was 21 (IQR 12.00-26.5), which was significantly higher compared with r-aPTH and non-PTH patients, both of which had a median RPQ score of 2 (IQR 0.00-7.0), p <0.001.

In multiple logistic regression analysis comparing pPTH with non-PTH patients, older age (OR 1.06, 95% Cl 1.01-1.13, p = 0.034) and insomnia after injury (OR 12.3, 95% Cl 2.79-54.13, *p* = 0.001) were independent risk factors for pPTH.

At one year, 4 patients (24% of pPTH patients) still suffered from headache.

### Psychiatric profile

In SCID-I interviews, psychiatric disorders were diagnosed in 7/17 (41%) of pPTH, 8/35 (22%) of aPTH, and 7/35 (20%) of non-PTH patients. The distribution of different psychiatric disorders did not differ between groups. The most common disorders were acute and current depression, panic disorder, and social anxiety disorder.

### Return to work

RTW at three months was documented for all patients who were either employed or full-time students before trauma (*n* = 91, including 6 students).

At three months, pPTH and r-aPTH patients differed significantly in the rate of return to full-time work (69% vs. 94%, *p* = 0.025). Similar difference was found between pPTH and non-PTH patients (69% vs. 93%, *p* = 0.035) (Table 4). At 12 months follow-up, all except one patient with pPTH had returned to work.

**Table 4:** Return to work at three months, comparison between pPTH and r-aPTH patients and between pPTH and non-PTH patients

Variable	pPTH *n* = 16 n (%)	r-aPTH *n* = 35 n (%)	p value (pPTH vs. aPTH)	Non-PTH n = 40 n (%)	*p* value (pPTH vs. non-PTH)
Full RTW	11 (68.8)	33 (94.3)	**0.025**	37 (92.5)	**0.035**
Partial RTW	3 (18.8)	0	**0.027**	3 (18.8)	0.338
Any RTW ^a^	14 (87.5)	33 (94.3)	0.581	40 (100)	0.078

Dichotomous variables: Pearson Chi–square test

^a^ Including both full- and partial RTW

*r-aPTH *recovered acute post-traumatic headache; *pPTH* persistent post-traumatic headache; *RTW* return to work

### Characteristics of headache in pPTH patients

Three out of 17 pPTH patients reported migraine-like headache: it was pulsating, severe, unilateral, and aggravated by physical activity, and rest eased the pain. In addition, concurrent nausea and sensitivity to light occurred. One pPTH patient with migraine-like headache also had tension-type headache. Four of 17 patients reported tension-type headache: the headache was blunt, pressure-type, waved, and rose from the neck to the whole head. There was no sensitivity to sound or light and no nausea or vomiting. One patient of 17 reported facial pain that resembled trigeminal neuralgia. 4/17 patients reported auditory symptoms such as tinnitus or buzz-like sounds and 8/17 patients reported sensitivity to light. For the remaining 5/17 patients, the neurologist had mentioned headache but not described the phenotypes, which thus remain unspecific.

## Discussion

Insomnia after injury increased risk for both aPTH and pPTH in this cohort of mTBI patients. Other risk factors for aPTH were headache at ED, other pain, and vertigo. Older age was a risk factor for pPTH. At three months, pPTH patients had a lower full-RTW rate than non-PTH patients.

In our study, the frequency of PTH was comparable to that in a recent study by Yilmaz and colleagues [10], with over half of patients having aPTH and nearly a quarter of them developing pPTH. In previous studies, history of headache and headache at ED have been identified as risk factors for PTH [10, 17, 25] being in line with our findings. Female gender was not associated with risk for PTH in our study, which may be explained by the lower number of patients than in previous studies [10, 17].

Pain other than headache after TBI was detected to be a risk factor for aPTH in our study. Lieba-Samal and colleagues similarly showed that other comorbid chronic pain, which was mostly musculoskeletal pain, was more frequent in aPTH patients compared with non-PTH (p = 0.05) [25]. Ashina and colleagues showed in a recent study that mTBI patients with pPTH rarely suffered from prior chronic pain (11 out of 100 subjects), but in a further 12-month follow-up, a notable proportion of pPTH patients reported frequent occurrence of post-trauma neck pain (78%) and low back pain (37%) [14].

Poor sleep quality and fatigue are some of the most common complaints after TBI and even more frequent among mTBI patients than among those with more severe TBIs [26]. A recent review analyzed the interaction between post-traumatic sleep disturbances and PTH [18]. A bi-directional relation was found: PTH was a risk factor for disrupted sleep, and post-traumatic sleep disruption caused more headaches. Disturbances of sleep were also highlighted in our study population, with aPTH patients suffering significantly more from pre- and post-injury insomnia compared with non-PTH patients. Post-traumatic insomnia was identified as a risk factor for both aPTH and pPTH. The mechanisms by which disrupted sleep and insomnia may worsen PTH remain unclear and need more research in the future.

Voormoolen and colleagues found that post-concussion symptoms (including PTH) were slightly more often reported by those mTBI patients who had trauma findings in brain imaging, that is complicated mTBI [27]. The authors stated that complicated mTBI was eventually only a weak indicator for post-concussion symptoms. Further, in a recent cohort study by Nordhaug and colleagues, pathological imaging findings in brain CT or MRI predicted aPTH, but not pPTH [28]. In our study, trauma findings in brain MRI did not significantly differ between pPTH and aPTH patients. Previous studies and our findings indicate that pathological brain imaging findings are not likely an essential risk factor for PTH.

The negative impact of PTH to RTW was previously studied by Dumken et al. where PTH intensity was measured by using the 0-10 numeric rating scale (NRS) with 0 meaning no headache and 10 the worst imaginable headache pain intensity [29]. The authors reported that each unit increase in headache pain intensity on the NRS reduced the odds of RTW by more than 50%. In a study by Stulemeijer and colleagues, low levels of post-concussion symptoms indicated good recovery and RTW [30]. In line with these studies, we identified that our pPTH patients were less likely to return to full-time work at three months compared with aPTH- and non-PTH patients [9, 10].

Previous studies have indicated increased psychiatric burden in PTH patients [10]. A recent case-control study showed that frequency of depression and anxiety, measured by Beck’s Depression Inventory and the State-Trait Anxiety Inventory, was higher among PTH patients than among patients with migraine or healthy controls [31]. In the present study, the frequency of psychiatric disorders did not differ between the PTH groups. This association should, however, be restudied in a larger cohort of PTH patients.

The phenotype of pPTH is unspecific, but often resembles migraine headaches [14]. In our study, the headache phenotypes were diverse, mostly unclassified type, tension type, or migraine-like headaches. The treatment of pPTH is challenging and multidisciplinary approach is needed, including preventive medications. Novel treatments like CGRP monoclonal antibodies might be an option in the future [16].

Strengths of our study are a well-defined cohort with several follow-up time-points, comprehensive brain imaging, and headache data from the ED.

As a limitation, the cohort is fairly small with many dropouts. Thus, the cohort may not be representative of all PTH patients. A second limitation is a lack of a systematic interview of the headache features at clinical neurological outpatient visits, but we used all the headache data available from the patient records. Thirdly, we did not have questionnaires to evaluate psychological burden and quality of life. However, we performed an extensive SCID-I interview.

## Conclusions

Insomnia after injury was associated with increased risk for both aPTH and pPTH in this cohort of mTBI patients. Further, other pain, headache at the ED and vertigo were risk factors for aPTH. With this we conclude that concomitant stress factors (e.g. insomnia and other pain) that are known to trigger also primary headache disorders play a key role in the development of PTH. pPTH and its negative impact to RTW was seen in our study population, highlighting the burden of this secondary headache disorder. Better understanding of the pathophysiology of pPTH is essential and multidisciplinary treatment approach is recommended.

## Data Availability

The data supporting the findings of this study are available from the corresponding author upon reasonable request.

## Abbreviations

aPTH: acute post traumatic headache
CGRP: calcitonin gene-related peptide
CT: computed tomography
ED: emergency department
GCS: Glasgow Coma Scale
ICHD: The International Classification of Headache Disorders
IQR: Interquartile range
MRI: Magnetic resonance imaging
mTBI: mild traumatic brain injury
PTH: post traumatic headache
pPTH: persistent post traumatic headache
RPQ: Rivermead post-concussion symptom questionnaire
RTW: return to work
SCID-I: Structured Clinical Interview for DSM-IV Axis I disorders
SD: standard deviation
TBI: traumatic brain injury
WHO: World Health Organization
